# Local Loperamide Injection Reduces Mechanosensitivity of Rat Cutaneous, Nociceptive C-Fibers

**DOI:** 10.1371/journal.pone.0042105

**Published:** 2012-07-25

**Authors:** Matthias Ringkamp, Michael Tal, Timothy V. Hartke, Matthew Wooten, Alvin McKelvy, Brian P. Turnquist, Yun Guan, Richard A. Meyer, Srinivasa N. Raja

**Affiliations:** 1 Department of Neurosurgery, School of Medicine, Johns Hopkins University, Baltimore, Maryland, United States of America; 2 Department of Anatomy and Cell Biology, Faculty of Dental Medicine, The Hebrew University-Hadassah, Jerusalem, Israel; 3 Department of Anesthesiology and Critical Care Medicine, School of Medicine, Johns Hopkins University, Baltimore, Maryland, United States of America; 4 Department of Mathematics and Computer Science, Bethel University, St. Paul, Minnesota, United States of America; University of Cincinnatti, United States of America

## Abstract

Loperamide reverses signs of mechanical hypersensitivity in an animal model of neuropathic pain suggesting that peripheral opioid receptors may be suitable targets for the treatment of neuropathic pain. Since little is known about loperamide effects on the responsiveness of primary afferent nerve fibers, *in vivo* electrophysiological recordings from unmyelinated afferents innervating the glabrous skin of the hind paw were performed in rats with an L5 spinal nerve lesion or sham surgery. Mechanical threshold and responsiveness to suprathreshold stimulation were tested before and after loperamide (1.25, 2.5 and 5 µg in 10 µl) or vehicle injection into the cutaneous receptive field. Loperamide dose-dependently decreased mechanosensitivity in unmyelinated afferents of nerve-injured and sham animals, and this effect was not blocked by naloxone pretreatment. We then investigated loperamide effects on nerve conduction by recording compound action potentials *in vitro* during incubation of the sciatic nerve with increasing loperamide concentrations. Loperamide dose-dependently decreased compound action potentials of myelinated and unmyelinated fibers (ED50 = 8 and 4 µg/10 µl, respectively). This blockade was not prevented by pre-incubation with naloxone. These results suggest that loperamide reversal of behavioral signs of neuropathic pain may be mediated, at least in part, by mechanisms independent of opioid receptors, most probably by local anesthetic actions.

## Introduction

The treatment of neuropathic pain continues to be a challenge with less than a third of patients obtaining satisfactory relief from presently available drugs [1]. Although opioids have been recommended as second line therapy for neuropathic pain, the beneficial effects of chronic use of systemic and intrathecal opioids in the treatment of neuropathic pain remains controversial [2]–[4]. Initial reports suggested that patients with cancer pain, including those with a neuropathic component, might benefit from treatment with chronic intrathecal morphine [5], [6]. However, subsequent studies implied that neuropathic pain may be resistant to opioids [7], [8]. More recent controlled trials with oral opioids by us [9] and other investigators [10]–[12] and case studies of intrathecal opioid treatment of neuropathic pain [13]–[15] demonstrate good pain relief even after long-term administration. However, the required doses of opioids may be higher than for the treatment of acute nociceptive pain, and side effects, such as sedation and cognitive dysfunction, associated with high doses of opioids limit their usefulness [9]. Additionally, concerns regarding addiction and the potential for abuse of this class of drugs have limited its widespread acceptance and use.

Traditionally, the analgesic effects of opioids were considered to result from their actions on the CNS. However, a growing body of evidence indicates that a significant part of the analgesic effects of opioids, after tissue injury and inflammation, is mediated by peripheral opioid receptors [16], [17]. Recent behavioral studies by us and others have indicated that systemic or intraplantar administration of loperamide, a peripherally acting µ-opioid receptor (MOR) agonist, leads to reversal of hyperalgesia in an animal model of neuropathic pain [18]–[20]. Evidence from these studies suggested that an important site of action for loperamide was in the tissues of the paw affected by the nerve injury. We postulated that the anti-hyperalgesic effects of loperamide were due, at least in part, to direct actions on peripheral nociceptive afferents.

In this study, we performed electrophysiological recordings of single nociceptive afferents that innervated the hindpaw in animals that had received a neuropathic lesion and in control animals. We investigated whether administration of loperamide into the cutaneous receptive field of nociceptive afferent fibers and large myelinated fibers affected their responsiveness to mechanical stimuli and whether such changes are mediated through an opioid mechanism.

## Methods

### Ethics Statement

Studies were approved by the Animal Care and Use Committee of the Johns Hopkins University as consistent with the National Institute of Health guide for the Use of Experimental Animals to ensure minimal animal use and discomfort.

### Spinal Nerve Lesion

A total of 55 male Sprague Dawley rats (250–300 g) were used in these studies. Animals were purchased from Harlan (Chicago, IL), housed in groups of 2, in cages with corn cob bedding, under a 12 hrs dark-light cycle with access to food and water ad libitum. Ligation of the left spinal nerve L5 was performed in 17 animals as described previously [21], [22]. Briefly, under isoflurane anesthesia (3% for induction, 1.5% for maintenance) a skin incision was made over the lumbar spine, and the back muscles on the left side of the animal were retracted. The lateral process of the L5 vertebra was removed, the spinal nerve L5 was isolated, and a segment of about 2 mm was removed. The muscle layer was closed with 4–0 vicryl suture and the skin incision over the lumbar spine was closed with wound clips. Concurrently with spinal nerve ligation (SNL), 12 animals received a sham surgery in order to perform the electrophysiological recordings (see below) under blinded condition. For sham surgery, animals simply received a skin incision over the lumbar spine that was closed with wound clips. Postoperatively, animals were monitored for an uneventful recovery up to 7 days.

### In vivo Electrophysiological Recordings

Electrophysiological recordings were done 7–10 days after nerve lesion or sham surgery. Animals were anaesthetized with pentobarbital i.p. (dose 50 mg/kg). The trachea was cannulated and animals were placed on a water-perfused heating blanket to maintain core temperature. ECG was recorded to monitor adequate depth of anesthesia. If noxious stimulation was accompanied by increases in heart rate ≥10%, supplemental doses (15 mg/kg) of pentobarbital were administered i.p. Animals were stabilized in a stereotactic frame. The left hind paw was extended and secured with the hairy side down in a block of clay thus allowing full access to the glabrous skin of the hind paw. An incision was made starting at the caudal edge of the pelvic bone and extended along the dorsal aspect toward the middle of the upper hind paw. The biceps femoris muscle was separated and the sciatic nerve was freed of connective tissue. A pool was formed by suturing the edges of the incised skin to a ring and filled with paraffin oil. The sciatic nerve was acutely crushed with blunt forceps just distal to the ischial foramen to block conduction of action potentials into the spinal cord. A small metal platform which also served as the return electrode was placed underneath the nerve, distal to the crush site, and the nerve sheath was carefully opened with watchmaker forceps. A silver wire that served as a recording electrode was placed over the splitting platform. Small nerve fiber bundles were cut at the proximal opening of the nerve sheath and carefully pulled away from the nerve stem using watchmaker forceps. Nerve bundles were teased into smaller filaments which were placed on the wire electrode to record orthograde neuronal activity. Filaments were teased into smaller filaments until activity from single, nerve fibers could be recorded.

Afferents innervating the hind foot were first activated by applying electrical stimuli (up to 80 mA, 1 ms duration) through two needle electrodes that had been inserted along the medial and lateral glabrous/hairy skin border of the foot. If neuronal activity from myelinated or unmyelinated fibers was present at the recording electrode, the glabrous skin of the paw was probed by applying pressure with a small, blunt tip of a glass rod. After locating the receptive field of a single unmyelinated nerve fiber, the borders of the receptive field were carefully mapped with a von Frey hair delivering a pressure of 6 bar (8 g). Responsive skin sites were marked on the skin with a permanent marker. Single unit activity was assumed if action potentials of similar shape were elicited from multiple spots, and if only a single nerve fiber could be activated when transcutaneous electrical stimuli were applied to the receptive spots. Using a slightly suprathreshold von Frey hair, the most sensitive spot within the cutaneous receptive field was located and marked on the skin, and experimental protocols (see below for details) were started. At the end of the *in vivo* electrophysiological recordings animals were sacrificed with an intravenous overdose of pentobarbital (100 mg/kg).

### Experimental Protocols

#### Unmyelinated afferents

After a 2 minute stimulus free interval, the mechanical von Frey threshold was determined at the most sensitive spot with a series of von Frey hairs exerting an increasing pressure. The smallest von Frey hair producing a response in two out of four applications that were separated by at least 15 sec after a response was defined as threshold. To measure the responsiveness to suprathreshold mechanical stimuli, an 8 g von Frey hair was then applied for 3 s and followed by a second application 30s later. After a 4 minute stimulus free interval, von Frey threshold and responsiveness to the suprathreshold mechanical stimulus was retested at least once or until stable baseline values were observed prior to administration of drugs. Von Frey threshold and responses to suprathreshold von Frey stimulation were re-assessed 5 minutes after drug injection, at the earliest, or after injection induced activity had subsided. Sensory testing was performed again 15 min after drug application. In experiments in which the responsiveness of the afferent under study was not changed after the first drug administration, another injection with the same or a higher dose was performed. Loperamide was injected into the receptive field at doses of 1.25, 2.5, 5 µg (injection volume 10 µl). In some of the initial *in vivo* experiments, the effect of vehicle and loperamide was tested in a blinded fashion. However, since vehicle did not have an effect (see results) only loperamide was injected in later experiments.

#### Myelinated fibers

As loperamide affected the mechanosensitivity of nociceptive afferents in a similar manner in nerve-lesioned and sham operated animals (see below for details), we also investigated, in unlesioned animals (n = 4), loperamide effects on the mechano-responsiveness of large myelinated fibers (conduction velocity 24.6±2.4 m/s, n = 10), which showed a slowly adapting response to mechanical stimulation with von Frey hairs. The experimental protocol for myelinated fibers was identical to that used for unmyelinated fibers, except that the effect of only one loperamide dose (5 µg in 10 µl) was tested.

#### Naloxone pretreatment experiments

As loperamide had similar effects in unmyelinated fibers of nerve-lesioned and sham operated animals (see below for details), we investigated if these effects were mediated through an opioid-dependent mechanism in a series of experiments in non-lesioned animals (n = 8). In these experiments, unmyelinated afferent nerve fibers were identified as described above. After assessing mechanical thresholds and responses to suprathreshold stimulation, cutaneous receptive fields of afferents were injected with naloxone (4 µg in10 µl or 80 µg in 20 µl), 5 min after which the viability of the afferent was tested by stimulation at the receptive field with an 8 g von Frey hair. Loperamide (5 µg in 10 µl) was then injected. Similar to the first series of experiments, mechanical thresholds and responses to suprathreshold stimuli were re-assessed 5 min after loperamide injection. Since low doses of naloxone (4 µg in10 µl) failed to prevent loperamide effects in a small number of experiments (n = 3), the receptive fields in the majority of afferents (n = 10) was injected with a high dose of naloxone (80 µg) in a volume of 20 µl to ensure that the pretreatment area would cover the loperamide injection area.

### In vitro Electrophysiological Recordings

We investigated the effect of loperamide on the conduction of peripheral nerve fibers *in vitro*. Unlesioned rats (n = 14) were euthanized with an overdose of pentobarbital (100 mg/kg, i.p.), and the sciatic nerves were harvested en bloc starting just distal to the lumbar plexus and including the tibial nerve at the heel. All other branches were cut. The nerves were transferred to an *in vitro* recording set up consisting of an organ bath and a recording chamber as described previously [23], [24]. Within the organ bath, the nerve was superfused with synthetic interstitial fluid (SIF) consisting of (in mM) 107.7 NaCl, 3.48 KCl, 0.69 MgSO4, 26.2 NaHCO3, 1.67 NaH2PO4, 1.53 CaCl2, 9.64 sodium gluconate, 5.5 glucose, and 7.6 sucrose. SIF was continuously bubbled with a mixture of 95% O2 and 5% CO2 to obtain a pH of 7.4. A roller pump (Gilson, model M312) was used to control the fluid of SIF at a rate of 750 ml/hr from the reservoir through a heat exchanger to the organ bath. The heat exchanger was used to raise the temperature of the SIF to 32°C. The tibial nerve was threaded through a hole from the organ bath into a mineral-oil-filled recording chamber containing a small glass mirror at the bottom that served as a splitting platform. After sealing the hole between both chambers with petroleum jelly and removing epi- and perineurium, the nerve was teased into smaller bundles until C fiber activity could be recorded in response to electrical stimuli applied at the sciatic nerve in the organ bath. We used this preparation instead of more conventional preparations usually used for compound action potential recordings, because it allowed easy application of drugs over a relatively long nerve length (70–80 mm) and because neuronal activity in single C fibers can be easily monitored.

To facilitate drug access to peripheral nerve fibers, the epineurium was stripped off the sciatic nerve. A Plexiglas ring (volume 1 ml, ID: 7 mm) with two groves at the bottom to accommodate the sciatic nerve was placed over the nerve and served as a drug application well. The well was sealed from the surrounding organ bath by petroleum jelly. Electrical stimuli (0.1 ms duration) of constant current (Digitimer DS7A, Hertfordshire, UK) were applied through a suction electrode placed outside the ring at the end of the de-sheathed sciatic nerve. After a filament containing C-fibers had been isolated, as indicated by neuronal activity recorded at a long conduction latency following electrical stimulation, electrical thresholds for A- and C-fibers and saturation intensities for their corresponding compound action potentials (CAP) were determined. For the remainder of the experiment, current intensity was set 1.5 x above saturation threshold for C-fibers to produce maximum stimulation of nerve fibers, and stimuli were applied continuously every 10 s (0.1 Hz).

In experiments investigating the effect of loperamide on conduction of myelinated and unmyelinated fibers, the sciatic nerve was first incubated with SIF for 10 min to establish baseline CAPs, followed by 20% (isotonic) CDEX, and then increasing concentrations of loperamide identical to those used in *in vivo* recordings (2.5, 5 and 10 µg/10 µl). SIF, CDEX and each loperamide concentration was applied for 10 min and was followed by a 10 min wash out with SIF to minimize carry forward effects and to evaluate recovery of CAPs. During each 10 min application period, the solution in the well was refreshed after 5 min. Following the washout after the highest loperamide dose, lidocaine (0.2%) was applied to the preparation until conduction in nerve fiber ceased. The remaining signal under lidocaine was used to quantify the stimulus artifact and to assess the contribution of the electrical noise to the signal (see Data analysis of more details).

In a second series of experiments we tested if naloxone pretreatment can block the observed loperamide effects on neuronal conduction. After establishing baseline CAPs during incubation with SIF (10 min), the nerve was first incubated with naloxone (4 µg/10 µl) for 5 min to pre- load the preparation, followed immediately by loperamide (5 µg/10 µl) for 10 min. After wash out (10 min), a second incubation with loperamide (5 µg/10 µl, 10 min) followed, after which lidocaine (0.2%) was applied. In contrast to the *in vivo* experiment, we did not use a higher concentration of naloxone (40 µg/10 µl) as this decreased CAPs of myelinated and unmyelinated fibers in pilot experiments. Furthermore, we did not co-apply naloxone with loperamide in these experiments, since CDEX in the loperamide solution could potentially interfere with naloxone action. Therefore and to resemble a protocol similar to the *in vivo* experiments, we pre-incubated the nerve with naloxone to saturate opoid receptors in the tissue prior to loperamide incubation. Although we cannot exclude some washout of naloxone during incubation with loperamide, such washout should be small as it only depends on passive diffusion in the *in vitro* preparation.

### Drugs

Loperamide (Sigma, St. Louis, IL) stock solution (4 mg/ml) was prepared in isotonic (20%) cyclodextrin, (CDEX, Sigma, St. Louis, IL) and further diluted with vehicle to achieve appropriate concentrations. Naloxone hydrochloride was purchased from Abbott Laboratories (Chicago, IL) or Sigma (St Louis, MO). In *in vivo* experiments, injections were administered with 28 1/2 G Lo-dose syringes (Becton Dickinson, Franklin Lakes, NJ). For *in vitro* experiments, naloxone was dissolved in SIF, and loperamide and naloxone solutions were kept at 30°C to minimize temperature effects on nerve conduction.

### Data Collection and Data Analysis

Neuronal signals were differentially amplified, filtered, digitized and stored on a personal computer using a data acquisition board and custom made data analysis and software system (DAPSYS, Brian Turnquist, Bethel University, St Paul, MN; see www.dapsys.net). Action potentials and other events were time stamped such that manipulations and neuronal activity could be correlated in time.

Von Frey thresholds and number of action potentials evoked by suprathreshold mechanical stimulation were used for statistical analysis. A pre-injection von Frey threshold and pre-injection response to suprathreshold stimulation was calculated by averaging data from the last two trials for each test prior to injection. Similarly, post injection values were calculated by averaging the data from two trials post injection. To avoid skin damage, the stiffest von Frey hair used in these studies was 26 g. If, following drug application, an afferent did not respond to this von Frey hair, the next higher von Fey hair was regarded as threshold (60 g). To compare responses across animals, post injection responses to suprathreshold stimulation were normalized by dividing the number of action potential post-injection by the number of action potentials observed prior to treatment.

The size of the A- and C- fiber compound action potentials recorded *in vitro* were analyzed as follows using Excel (Microsoft Office 2003). Six traces from the last minute of lidocaine treatment were averaged to generate a measure of the electrical noise present in each trace. This average noise was subtracted from every trace recorded in the 1^st^, 5^th^ and 10^th^ minute of each incubation period in order to remove small DC-offsets that otherwise would have contaminated the analysis and also to remove the stimulus artifact from the recorded signal so that the A-fiber signal could be properly analyzed. Following noise removal, the signal strength of each trace was determined using root-mean-square (RMS), with RMS  =  √∑ ((y_n_)^2^)/n). In order to assess the effect of loperamide separately for A- and C-fibers, RMS values were calculated separately for each class of fiber. For A fibers, RMS was calculated over the period of 1–15 ms after stimulation. For C-fibers, RMS was calculated over the period of 50–150 ms following stimulation. The RMS values for the six traces obtained in each minute were averaged, and this averaged data for 1^st^, 5^th^ and 10^th^ minute of each incubation period were analyzed. RMS values of the same incubation period varied considerably between experiments because we could not control, for example, the number of fibers contributing to the recorded signal. Therefore, in order to compare between different preparation, the RMS values were normalized by using the following formula: normalized RMS  =  (RMS_X_ –RMS_Lido_)/(RMS_SIF10min_- RMS_Lido_), where RMS_X_ is the average RMS of the 1^st^, 5^th^ or 10^th^ min of the different incubations/washout periods, RMS_SIF10min_ is the average RMS of the 10^th^ min under SIF incubation and RMS_Lido_ is the average RMS recorded under lidocaine. This normalized RMS was entered into statistical analysis.

Data were analyzed using STATISTICA 6.1 (StatSoft, Inc, Tulsa, OK). Data for mechanical thresholds were analyzed with Kruskal ANOVA followed by multiple comparisons of mean ranks between groups (Siegel & Castellano, 1988). Data at a given dose were tested for animal group differences with Mann Whitney U test. Data for the responsiveness to suprathreshold stimulation were normally distributed and therefore analyzed with parametric tests (ANOVA) followed by Scheffé test for post hoc comparison. Data from experiments in which the effects of loperamide on compound action potential were investigated were not normally distributed and therefore analyzed with Friedman ANOVA followed by Wilcoxon matched pairs test for post hoc testing. Data from compound action potential recordings in which the inhibitory effect of naloxone on loperamide induced effects were normally distributed and therefore analyzed with ANOVA, followed by paired t –tests.

## Results

### Electrophysiology

We recorded from a total of 49 unmyelinated, nociceptive afferents, 24 were recorded from lesioned and 25 from unlesioned animals. The effect of local loperamide injection on mechanosensitivity was tested in a total of 36 afferents, 24 of which were recorded in lesioned animals. In 13 unmyelinated afferents from unlesioned animals, the effect of naloxone pretreatment on loperamide-induced effects on mechanosensitivity was tested. All unmyelinated afferents had receptive fields in the glabrous skin. Conduction velocities of unmyelinated afferents were significantly slower in lesioned than in sham operated animals (0.59±0.03 m/s vs. 0.70±0.05 m/s, p<0.05, t-test for independent samples).

### Loperamide Increases Mechanical Thresholds and Decreases the Response to Suprathreshold Mechanical Stimuli of Unmyelinated C-fiber Afferents

**Figure 1:**
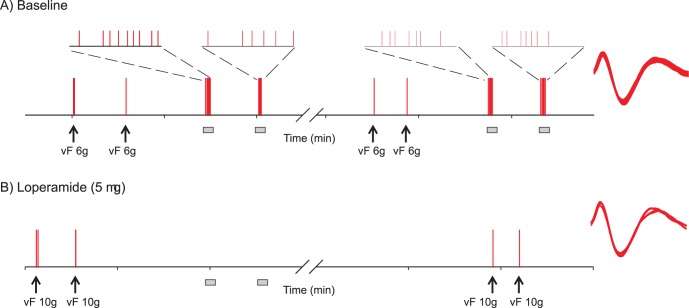
Specimen recording showing the effect of loperamide on the mechanosensitivity of an unmyelinated afferent (AE49.02C) recorded from a spinal nerve lesioned animal 9 days post injury. Vertical bars indicate occurrence of an action potential (AP). Arrows indicate von Frey hair application to determine mechanical threshold. Grey bars indicate tonic suprathreshold stimulation (5 s) with an 8 g von Frey hair. A) Responses to mechanical tests at baseline. In the two test sequences for baseline testing, the afferent responded to 2/2 trials of stimulation with a 6 g von Frey hair (but not to the next lower 4 gm von Frey hair, data not shown), and suprathreshold stimulation with 8 g von Frey hair induced a total of 15 and 14 action potentials in the first and second test sequence, respectively. Insets show occurrence of action potential recorded during suprathreshold stimulation at higher time resolution. On the right, all AP waveforms recorded during baseline testing are shown superimposed. B) Mechanical responses after injection of a cumulative 5 µg dose of loperamide (1.25 µg followed by 3.75 µg). The von Frey threshold increased to 10 g, and the afferent became unresponsive to tonic stimulation with 8 g. In the second test sequence, mechanical von Frey threshold was still 10 g. Responses to 8 g von Frey hair stimulation were not tested.

An example of the loperamide effect on the mechanosensitivity of unmyelinated afferents is shown in Figure 1. This afferent was recorded from a spinal nerve lesioned animal 9 days after injury. In both test sequences at baseline (panel A), each of the two applications of a 6 g von Frey hair (indicated by arrows) activated the unmyelinated afferent. Stimulation with smaller von Frey hairs failed to induce activity, and, therefore, 6 g was considered the mechanical threshold. Tonic suprathreshold stimulations with an 8 g von Frey hair (indicated by grey boxes) produced a total of 15 action potentials in the first test sequence and a total of 14 action potentials in the second. After a cumulative dose of 5 µg of loperamide had been administered in the receptive field (panel B), the afferent responded in both test sequences to stimulations with a 10 g von Frey hair (indicated by arrows) but not to stimulation with smaller von Frey hairs (not shown), i.e. the mechanical threshold had increased. In agreement with a threshold of 10 g, tonic stimulations with an 8 g von Frey hair (grey boxes) did not activate the afferent.

**Figure 2:**
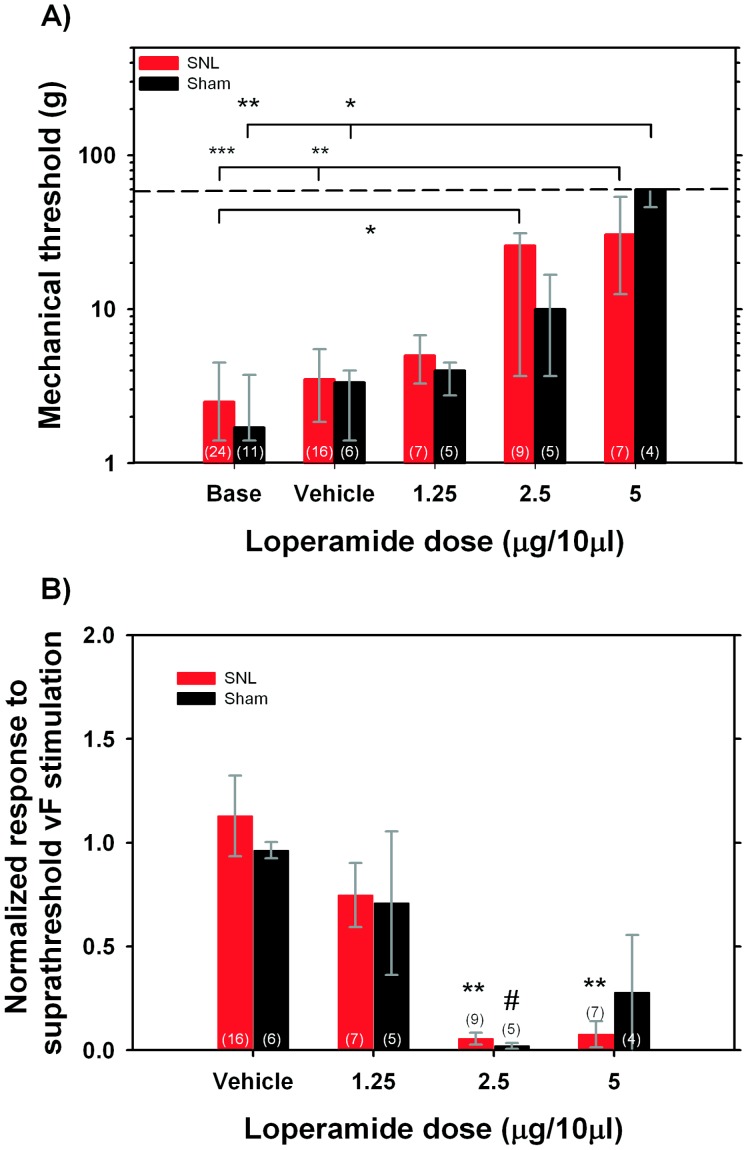
Loperamide decreases mechanical responsiveness of unmyelinated, nociceptive C-fibers. A) Loperamide dose-dependently increases mechanical thresholds in both sham operated and spinal nerve lesioned (SNL) animals. Kruskal Wallis ANOVA (p<0.01 in both groups) was followed in each group by multiple post-hoc comparisons between mean ranks. Significant differences are indicated as *p<0.05, **p<0.01, ***p<0.001. At no given dose was there a significant difference between SNL and sham group (Mann Whitney U test, n.s.). Medians, 75th and 25th percentile are plotted. Numbers of fibers studied in each group and dose are given in parentheses. Dashed line indicates threshold assigned to afferents unresponsive to 25 g vF hair. B) Loperamide dose-dependently decreases the responsiveness to suprathreshold mechanical stimuli in both animal groups (ANOVA lesion: F_(3,35)_ = 10.3, p<0.001; ANOVA sham: F_(3,16)_ = 4.5, p<0.05). Post hoc comparisons revealed significant differences between vehicle and different loperamide doses where indicated (Scheffe test; **p<0.01, *# p<0.05). Numbers in parentheses state the total number of fibers studied in each group and dose.

Mechanical thresholds prior to injection of loperamide did not significantly differ between nerve lesioned and sham operated animals (2.5 g [1.4–4.5 g] vs 1.7 g [1.4–3.75 g]). In both control and nerve injured animals, mechanical thresholds were significantly changed following loperamide injection (sham group: H_(4,31)_ = 16.5, p<0.01; lesion group: H_(4, 63)_ = 24.1, p<0.001). Following the injection of 5 µg of loperamide, mechanical thresholds in sham and nerve lesioned animals increased to 60 g [46]–[60] and 30.7 g [12.5–60] respectively, and these thresholds were significantly greater than thresholds at baseline or thresholds obtained following injection of vehicle (p<0.01 and p<0.05, see fig. 2A). While mechanical thresholds increased in both groups following the injection of smaller doses of loperamide (i.e., 1.25, 2.5 µg), these thresholds were significantly different from pre-injection thresholds only following the 2.5 µg dose, but not after the 1.25 µg dose. Similarly, mechanical thresholds were not changed following the injection of vehicle. Regardless of the loperamide dose, we did not observe significant differences in post injection mechanical thresholds between animal groups, i.e. loperamide had similar effects in sham-operated and nerve lesioned animals.

Baseline responsiveness to the suprathreshold mechanical stimulus (8 g von Frey hair for 3 s) did not differ significantly between sham and nerve lesioned animals (13 APs [6]–[19] vs 12 APs [7]–[14]). In order to compare the drug effects between animals, the response to suprathreshold stimulation was normalized to the baseline response in each animal. A two-way ANOVA with animal group (lesion, sham) and drug dose as main factors revealed that only drug dose had a significant effect (F_(3,51)_ = 11.7, p<0.001) but not animal group (F_(1,51)_ = 0.004, p = 0.95). To analyze the drug effect in more detail, we performed a separate ANOVA for each animal group. As can be seen from figure 2B, administration of loperamide inhibited the response, in a dose dependent manner, in both animal groups (lesion: F_(3,35)_ = 10.3, p<0.001; sham: F_(3,16)_ = 4.5, p<0.05). In the sham operated group, Scheffé comparisons between different doses revealed a significant difference between vehicle injection and the 2.5 µg dose of loperamide (p<0.05). In spinal nerve lesioned animals, the normalized responses following 2.5 and 5 µg of loperamide were significantly smaller than the responses seen following vehicle injection (p<0.01 for both doses). In control and lesioned animals, the smallest loperamide dose (1.25 µg in 10 µl) did not significantly change the response to suprathreshold stimulation.

### Naloxone Pretreatment does not Prevent Loperamide-induced Decrease in Mechanosensitivity

**Figure 3:**
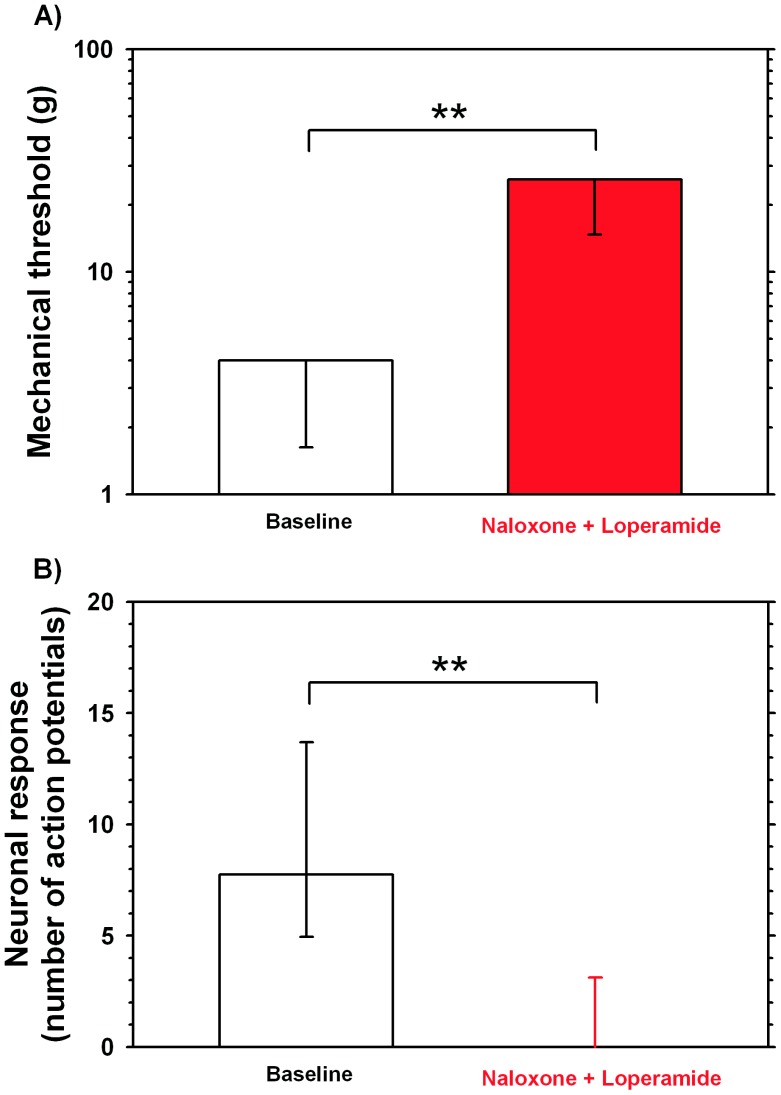
Naloxone does not block loperamide effects on mechanosensitivity of C-fibers. A) Naloxone pretreatment does not prevent the loperamide (5 µg/10 µl) – induced increase in mechanical thresholds of C-fibers. Naloxone was used either in a small (4 µg in 10 µl, n = 3) or a high dose (80 µg in 20 µl, n = 10). Medians and 25th percentile data are shown. 75th percentiles were identical to median value. (**p<0.01, Wilcoxon matched pairs, n = 13). B) Loperamide –induced decrease in response to suprathreshold mechanical stimulation, is not prevented by naloxone pretreatment. Loperamide and naloxone doses are identical to those used in A. Medians, 25th percentile and 75th percentiles are shown. (**p<0.01, Wilcoxon matched pairs).

To test if the observed loperamide effect on the mechanosensitivity of unmyelinated fibers were mediated by an opioid mechanism, we investigated if pretreatment with naloxone (either 4 µg in 10 µl (n = 3) or 80 µg in 20 µl (n = 10)) is able to prevent the inhibitory effects of loperamide in 13 unmyelinated afferents from unlesioned animals (n = 8). Baseline mechanical threshold prior to injection of naloxone were on average 3.23±0.5 g (median 4 g, 25^th^ percentile: 1.7 g, 75^th^ percentile 4 g). Following injection of loperamide, the average mechanical thresholds increased significantly to 19.5±2.3 g (median: 26 g, 25^th^ percentile: 15 g, 75^th^ percentile 26 g; p<0.01; Wilcoxon matched pairs, see Fig. 3A), and responses to suprathreshold mechanical stimulation with 8 g decreased significantly (p<0.01 Wilcoxon matched pairs, see Fig. 3B). Taken together, these data suggest that the inhibitory effects of loperamide on the mechanosensitivity of unmyelinated fibers may be mediated by a mechanism that is not dependent on opioid receptors.

### Loperamide does not Alter the Mechanosensitivity of Slowly Adapting Myelinated Afferents

**Figure 4:**
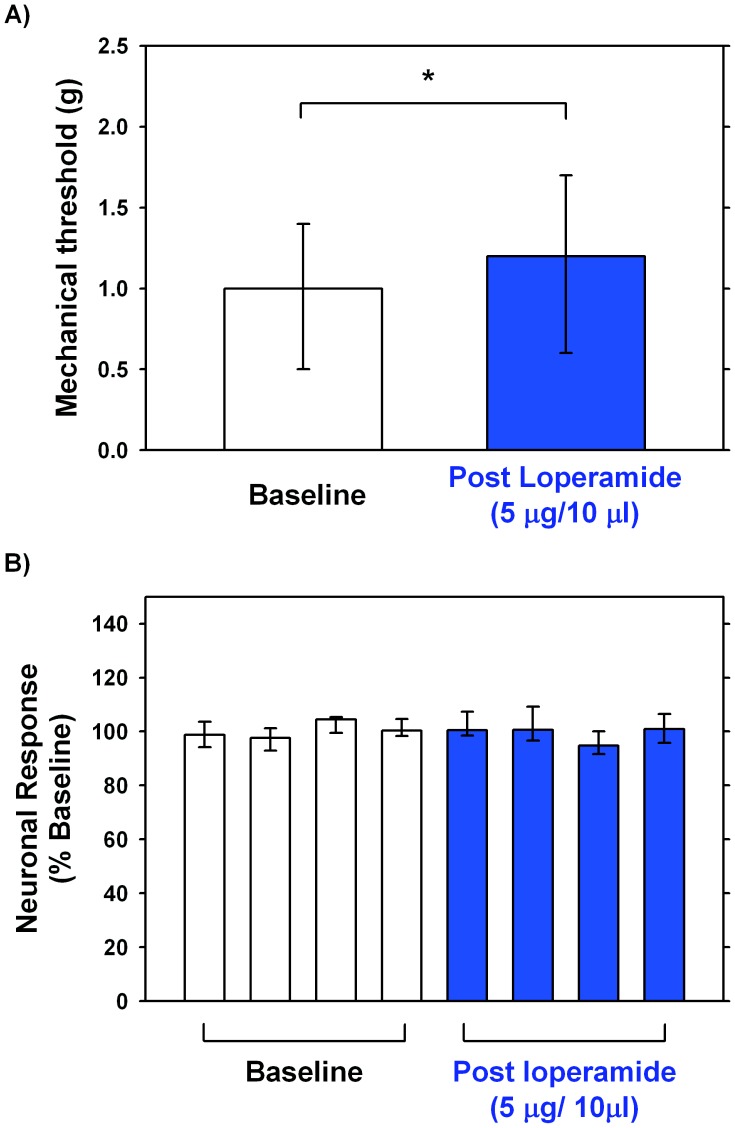
Effects of loperamide on myelinated low-threshold mechanoreceptors. An experimental protocol identical to that used in C-fibers was employed to investigate the effects of loperamide (5 µg/10µl) on the mechanosensitivity of slowly adapting Aβ- fibers (CV: 26.6 m/s. n = 10) innervating the glabrous skin of the hind paw. A) Loperamide increased mechanical von Frey thresholds significantly (*p<0.05, Wilcoxon matched pairs), but only slightly (median, before vs after: 1.0 g vs 1.2 g shown). Importantly, all afferents remained sensitive to mechanical stimulation. Medians, 25th percentile and 75th percentiles are shown. B). Loperamide (5 µg/10µl) did not significantly change the responses to suprathreshold stimulation (Friedman ANOVA, p: n.s., n = 10). To compare responses to suprathreshold stimulation (8 g) across fibers, each response in a given fiber was normalized by dividing it by each fiber’s grand average, i.e. the average of the responses in the 4 trials prior to injection of loperamide. These normalized data were then used for statistical analysis. Medians, 25th percentile and 75th percentiles are shown.

The effects of loperamide on the mechanosensitivity of large myelinated fibers was investigated in 10 fibers (conduction velocity 24.6±2.4 m/s) recorded from unlesioned animals. These afferents showed a slowly adapting response when stimulated with von Frey hairs. At baseline the average mechanical threshold in these afferents was 1.19±0.34 g. Following loperamide injection the mechanical threshold increased to 1.38±0.35 g which was significantly different from baseline values (Wilcoxon matched pairs, p<0.05; Fig 4A). As the effect of vehicle was not tested in myelinated fibers, we cannot rule out that this small change in mechanical threshold is due to a vehicle effect. To evaluate loperamide-induced changes to suprathreshold stimulation, each response (i.e. number of evoked action potentials) was normalized to the average response obtained during baseline testing. Normalized responses to suprathreshold stimulation did not significantly change after injection of loperamide (Friedman ANOVA, p<0.29; Fig 4B). Taken together these data show that loperamide (5 µg) has only a small, if any, effect on the mechanosensitivity of large myelinated fibers.

### Loperamide Blocks Conduction in Myelinated and Unmyelinated Fibers

**Figure 5:**
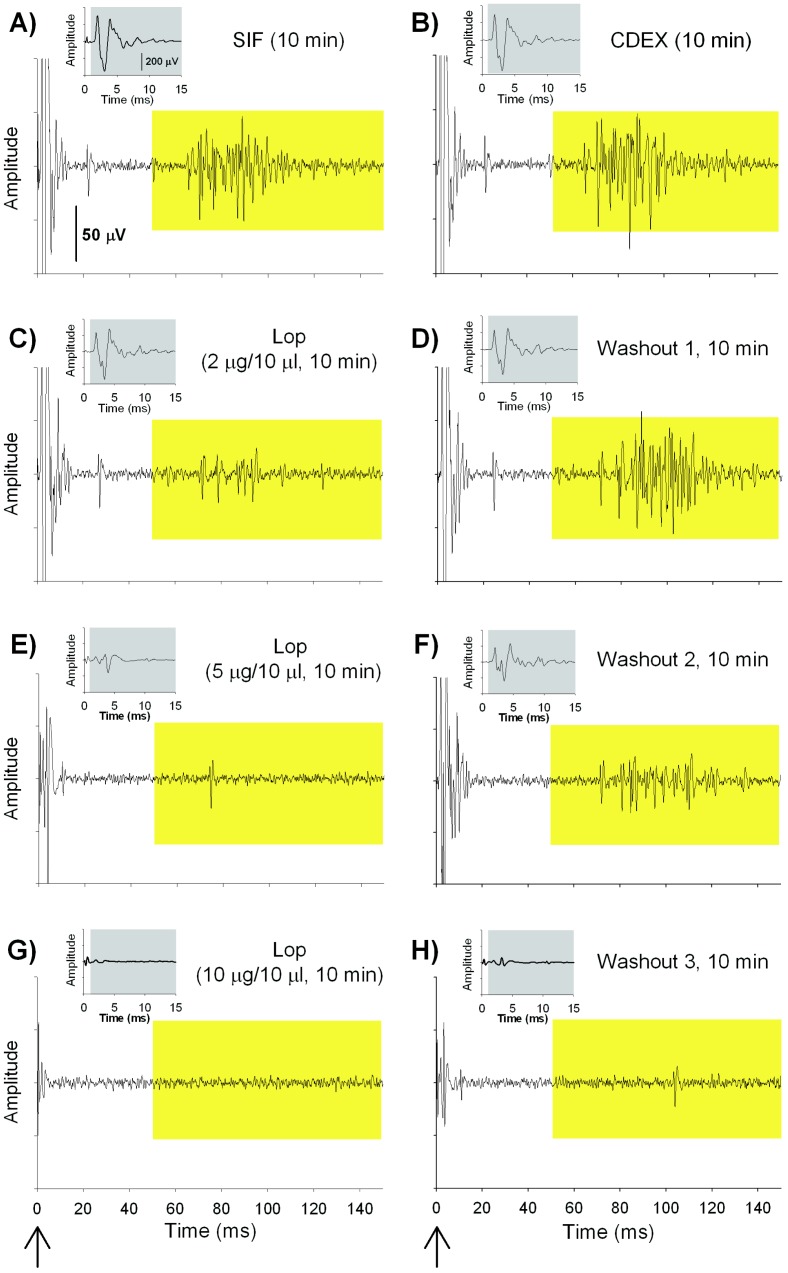
Effects of loperamide on tibial nerve compound action potential recordings. Examples (AF80.02C) of *in vitro* compound action potential (CAP) recordings from a small nerve bundle in the tibial nerve (arrow at bottom of figure indicates time of electrical stimulation). The size of the A-fiber CAP (grey box, insert) and C-fiber CAP (yellow box) decreased during incubation with loperamide in a dose dependent manner. During washout, the neuronal activity recovered except after the highest dose of loperamide. (A–H) The CAP recorded at different time points during the experimental protocol indicating the effects of different doses of loperamide.

Naloxone pretreatment did not prevent the loperamide-induced inhibition of mechanosensitivity in unmyelinated afferents, suggesting that loperamide produces its effect through non-opioid mechanisms. We wondered whether loperamide’s effects were not at the peripheral terminals where MORs are thought to exist but rather along the course of the peripheral nerve fibers. We therefore investigated, *in vitro,* the effects of different concentrations of loperamide (2, 5 and 10 µg/10 µl) on A- and C-fiber compound action potentials in the sciatic nerve. An example of such an experiment is shown in Fig. 5. As can be seen, increasing doses of loperamide decreased the electrically induced A- and C-fiber response. Importantly, responses in both fiber types recovered during wash out with SIF, except after the highest dose of loperamide (10 µg/10µl).

**Figure 6:**
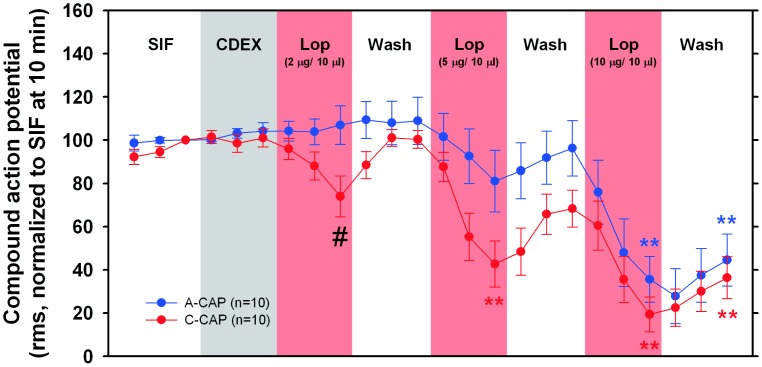
Loperamide produces a conduction blockade in A and C fibers in the peripheral nerve. The A-CAPs (blue circles) changed significantly with different incubation steps (Friedman ANOVA, p<0.001). After incubation with the highest loperamide dose, the A-CAP was significantly reduced compared to A-CAP under CDEX (10th min) (**p<0.01, Wilcoxon-matched pairs with correction for multiple testing). The C-CAP (red circles) also changed significantly during the course of the experiment (Friedman ANOVA, p<0.001). Under loperamide (5 and 10 µg/10 µl), the C-CAP significantly decreased compared to CDEX C-CAP (**p<0.01, Wilcoxon-matched pairs with correction for multiple testing). At the lowest loperamide concentration (2 µg/10 µl), the C-CAP was significantly smaller than the A-CAP (#p<0.05 Mann Whitney U test).

A summary of the data collected in these *in vitro* experiments are shown in Fig. 6. Incubation with loperamide caused significant changes in the C-CAP (Friedman ANOVA, p<0.001, Fig. 6). Although the C-CAP was reduced following prolonged incubation (10 min) with every loperamide concentration used, only during the incubation with higher concentrations (5 and 10 µg/10 µl) were the C-CAPs significantly smaller when compared to the C-CAP following 10 min incubation with CDEX (p<0.01, Wilcoxon matched pairs, corrected for multiple testing). In control experiments, in which the nerve was repetitively incubated with isotonic CDEX for 60 min, the C-fiber CAP showed a small but significant increase (Friedman ANOVA, p<0.001), and at the end of the protocol C-CAP was 136% (median: 123%, 25^th^ percentile: 97%, 75^th^ percentile: 158%, n = 10, data not shown), but this was not significantly different when compared to the C-CAP during the 10^th^ minute of the first incubation with CDEX.

Incubation with loperamide also caused significant changes in the A-CAP (Friedman ANOVA, p<0.001, Fig. 6). During the 10^th^ minute of incubation with loperamide (10 µg/10 µl), the A-CAP was reduced to 36% (median: 27%, 25^th^ percentile: 8.6%, 75^th^ percentile: 57%, n = 10), and this was significantly different from the A-CAP recorded during the 10^th^ minute of incubation with isotonic CDEX, the vehicle used for loperamide (p<0.01, Wilcoxon matched pairs, corrected for multiple testing). Following 10 min incubation with loperamide at 5 µg/10 µl the A-CAP size was significantly reduced to about 80%. In contrast to the C-CAP, the A-fiber CAP showed a small but significant decrease during repetitive incubation with CDEX in control experiments (Friedman ANOVA, p<0.001). At the end of these control experiments, the A-fiber CAP was decreased to 72% (median: 78%, 25^th^ percentile: 49%, 75^th^ percentile 91%, n = 11, data not shown), but this was not significantly different when compared to A-CAP during the 10^th^ min of the first incubation with CDEX.

**Figure 7:**
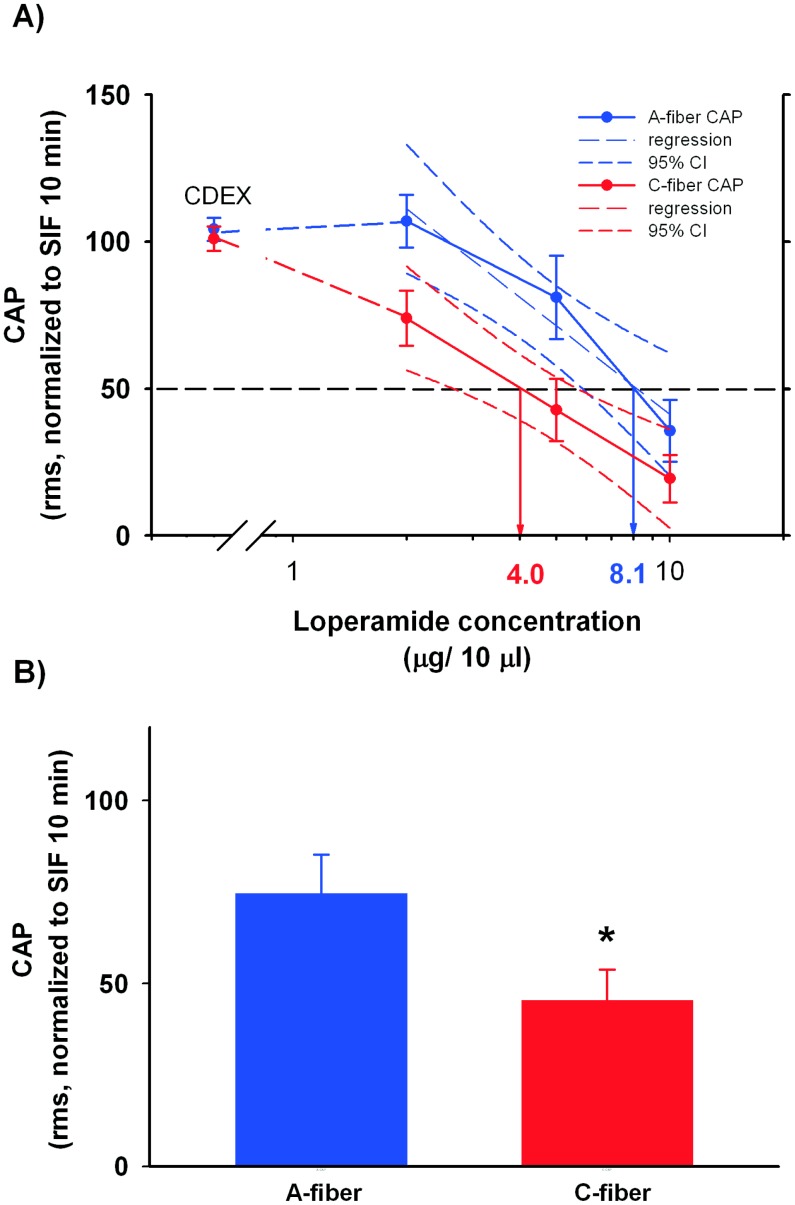
The conduction blockade by loperamide was dose-dependent. A) Dose response curves for the A- and C – CAPs were plotted, and regression analyses were performed for both to estimate the ED_50_. For the C-CAP (red symbols), the regression line and the dose response data completely overlap. The ED_50_ for C-CAP (4.0 µg/10 µl) is lower than the ED_50_ for A-CAP (8.1 µg/10µl), but the 95% confidence intervals overlap slightly. B) The average A-CAP during loperamide incubation was significantly larger than the average C-CAP (inset, paired t-test, p<0.05, n = 10), suggesting that A fibers are less susceptible to loperamide effects.

As shown in Fig. 6, following incubation with high concentrations of loperamide, the A- and C-CAP did not significantly differ. However, after 10 min incubation with the low loperamide concentration, the C-CAP was significantly smaller than the A-CAP (# p<0.05, Mann Whitney U test). We estimated the ED_50_ of loperamide for the A- and C- CAP from the corresponding regression lines of the dose- response curves as shown in Fig. 7. For the C-CAP, the ED50 was 4.0 µg/10 µl, whereas the ED50 for A-CAP was about 8 µg/10 µl. As illustrated in Fig. 7A, the confidence intervals of the 2 linear regression curves slightly overlapped, suggesting that the ED_50_ for the A- and C-CAP do not significantly differ. However, when we compared the average effect of loperamide across the different concentrations used (see Fig. 7B), the C-CAP under loperamide was significantly smaller than the A-CAP (p<0.05, Wilcoxon matched pairs). These finding suggests that A-fibers are less susceptible to the effects of loperamide than C-fibers.

### Naloxone does not Prevent the Loperamide-induced Decrease in CAPs

**Figure 8:**
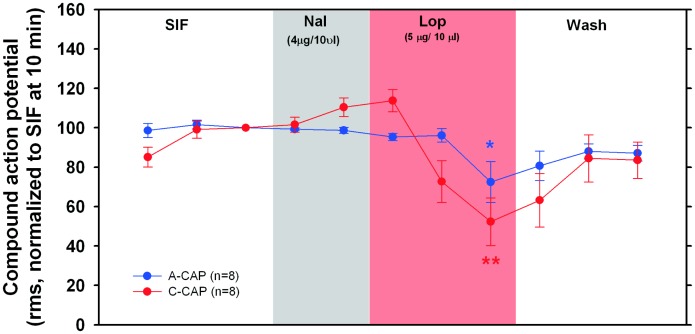
Naloxone does not prevent the conduction blockade effects of loperamide. The nerve was preincubated with naloxone (4 µg/10 µl) for 5 minutes followd by incubation with loperamide (5 µg/10 µl for 10 min). Preincubation with naloxone did not prevent loperamide induced changes of A- and C- CAPs (p<0.001, repeated measures ANOVA). Incubation with loperamide significantly reduced A-CAP and C- CAP compared to 5 min SIF incubation (**p<0.01, *p<0.05, paired t-test, n = 8).

To test if the impaired conduction of A-and C-fibers under loperamide incubation is mediated by opioid receptors, we pre-incubated the nerve segment to be exposed to loperamide with naloxone (4 µg/10 µl) for 5 minutes. Without washing, the nerve was then incubated with loperamide (5 µg/10 µl). As shown in Fig. 8, pre-incubation with naloxone did not prevent the decrease of the CAPs induced by loperamide. Both, A- and C- fiber CAPs changed significantly during the course of the experiment (A CAP: repeated measures ANOVA, F(9,63) = 5.02, p<0.001; C CAP: repeated measures ANOVA, F(9, 63) = 8.73, p<0.001). In the 10^th^ minute of incubation with loperamide (5 µg/10 µl), the A-CAP and C- CAP were significantly reduced to 72.4% ±10.4% (p<0.05, paired t-test) and 52.3% ±12.1% (p<0.01, paired t-test), respectively. These values were significantly different from the CAPs recorded at 5 min during incubation with SIF (A CAP, p<0.05; C-CAP, p<0.01; paired t-tests). Importantly, these values were not significantly different from those observed for loperamide incubation (5 µg/10 µl) without preceding naloxone incubation (Mann Whitney U test, n.s.). Therefore, it is unlikely that the loperamide effect on the conduction of myelinated and unmyelinated fibers is mediated by opioid receptors.

### Effects of Systemic Loperamide Injection

In 3 C fibers (2 fibers from 2 sham operated animals and 1 fiber from a nerve injured animal) and 2Aβ- and 1Aδ –fibers (from nerve lesioned animals) we studied the effect of systemic loperamide injection on the responsiveness to mechanical stimuli. Loperamide (10 mg/kg) was injected subcutaneously into the nape of the neck; this dose was shown in previous behavioral experiments to lead to an antihyperalgesic effect [18]. Tests were performed at 10 min intervals for up to 40 min after injection. Surprisingly, systemic loperamide did not have any obvious effect on mechanical thresholds or the responsiveness for suprathreshold stimuli. The proportion of C-fibers that were affected by systemic loperamide (0/3) was significantly lower than the proportion that were affected by intradermal loperamide at the 5 µg/10 µl dose (11/11; χ^2^ ≤0.001).

## Discussion

In this study, injection of loperamide in the cutaneous receptive field decreased the response of unmyelinated nociceptors to mechanical stimuli. Unexpectedly, this effect was seen in both nerve lesioned and in control animals. Naloxone pretreatment did not prevent loperamide induced inhibition of mechanosensitivity in unmyelinated afferents. In addition, loperamide produced a dose-dependent conduction block in myelinated and unmyelinated peripheral nerve fibers which could not be prevented by pretreatment with naloxone. An opioid-receptor independent mechanism may therefore contribute to the reversal of mechanical hyperalgesia observed previously in an animal model of neuropathic pain.

We had previously reported the development of spontaneous activity in uninjured L4 fibers following L5 spinal nerve injury [25], [26]. In the present study we did not observe spontaneous activity. As the previously observed spontaneous activity was low (median: 7 action potentials in 5 min), we may have missed low frequency activity in the current study, because we did not provide a 5 min observation period at the beginning of our recordings but focused instead on stimulus evoked responses. Others have previously reported the sensitization of unlesioned L4 C- fibers to mechanical and thermal stimuli [27], but we did not find such sensitization to mechanical stimuli in this study. Methodological differences (i.e. application of von Frey hairs by hand vs micromanipulator; suprathreshold stimuli 8 g vs 99 g) may, however, account for these different findings.

### MORs and Primary Afferents

Unmyelinated cutaneous nerve fibers express MORs on their peripheral terminals [28]. The role of these receptors under normal physiologic conditions is unclear, as the responsiveness of cutaneous unmyelinated nociceptive afferents in monkey and rat was not altered following systemic doses of morphine known to produce analgesia in man [29], [30]. However, inhibition of nociceptive afferents by opioids has been observed under inflammatory conditions. For example, the spontaneous discharge in nociceptive afferents innervating the inflamed knee joint was significantly inhibited by morphine treatment [31]. This effect was reversible by naloxone. In addition, spontaneous activity in cutaneous nociceptive afferents following UV irradiation was blocked when opioids were applied to their receptive fields [32]. Furthermore, in inflamed but not in normal skin, morphine reduced mechanical and heat-evoked responses in nociceptors [33].

### Peripheral MORs and Neuropathic Pain

Peripheral opioid analgesia has been demonstrated in experimental models of inflammatory pain [20], [34], [35], and in clinical studies, e.g., intraarticular administration after arthroscopy [36]. Possible underlying mechanisms include increased synthesis of MORs in the DRG and enhanced axonal transport of opioid receptors to the periphery [34]. Recent reports suggest that a similar peripheral opioid receptor-mediated analgesia may also occur in neuropathic pain. Morphine superfused over the ligature site in the chronic constriction injury (CCI) model reversed thermal and mechanical hyperalgesia in a dose-related, naloxone-sensitive fashion [37]. Other unblinded studies report that intraplantar injections of morphine in the nerve-injured, but not contralateral paw produce a dose-related elevation of paw withdrawal and vocalization thresholds to mechanical stimuli in CCI and SNL models [38], [39]. In addition, the antihyperalgesic effects of systemic morphine were attenuated by intraplantar injection of a peripherally acting MOR antagonist. Also intraplantar injection of nmol doses of selective MOR agonists, such as DAMGO, showed a reduction in hyperalgesia in the CCI model [40].

### Peripherally Acting MOR Agonists and Antagonists

Two peripherally selective MOR agonists have been well studied. These include the quaternary derivative of morphine, N-methyl morphine [41], [42] and the antidiarrheal agent, loperamide. Topical or local administration of loperamide results in antihyperalgesia without the side effects associated with systemic opioid administration. Local injection of loperamide in the inflamed, but not the contralateral, paw attenuates Freund’s adjuvant-induced hyperalgesia with a potency comparable to that of morphine [43]. Similar effects have been reported with a topical administration of 5% loperamide cream in a model of burn-induced hyperalgesia [44]. The behavioral effects of loperamide were observed in the absence of measurable concentration of the drug in blood. Our recent behavioral studies demonstrate that loperamide is also effective in attenuating mechanical allodynia in the SNL model of neuropathic pain [18].

The potential advantages of a peripherally acting opioid agonist that attenuates neuropathic pain are the lack of CNS side effects and the significantly lower potential for addiction and drug abuse. Loperamide is more selective for the MOR subtype than for other opioid receptor subtypes and does not penetrate the brain in appreciable amounts [41]. Pharmacokinetic studies show minimal accumulation of drug in the brain following IV injections [45]. After oral administration of loperamide, the bulk of the drug remains in the gastro-intestinal tract and is excreted mostly as unchanged drug [46]; hence, the oral route is not likely to be associated with analgesic effects. The peripheral selectivity of loperamide is thought to be secondary to its lipophilicity and ability to serve as a substrate for the multi drug resistant (MDR) transporter [45], [47]. Clinical studies have shown that loperamide does not possess abuse potential or dependence liability [48], [49]. A potential disadvantage with peripherally acting opioids is that they are likely to share the gastro-intestinal side effects common to all opioids, such as reduced gut motility.

### Loperamide Effects and Mechanisms of Action

Loperamide dose-dependently reduced the mechanosensitivity of unmyelinated cutaneous afferents of nerve-lesioned and normal animals. Because previous studies did not find opioid effects in normal tissue, and to elucidate further the mechanisms underlying the observed loperamide effects, naloxone pretreatment was used in an attempt to prevent loperamide effects. Naloxone pretreatment of the receptive field failed to inhibit the loperamide induced decrease in mechanosensitivity of unmyelinated fibers. Furthermore, in *in vitro* experiments, loperamide dose-dependently decreased CAP of myelinated and unmyelinated fibers in the desheathed sciatic nerve, a finding consistent with previous reports [50], [51]. At a dose of 5 µg/10 µl,loperamide markedly inhibited the mechanosensitivity in unmyelinated afferents, but it did not reduce mechanosensitivity of slowly adapting myelinated fibers. Consistent with this finding, this loperamide concentration reduced the A-fiber CAP to ‘only’ about 80%, whereas C-fiber CAP was reduced by more than 50%. We do not know what fiber types contributed to the 20% reduction of A-CAP under 5 ug/10 ul of loperamide, but it may be caused mainly by a conduction loss in motor fibers and muscle afferents and not myelinated afferents innervating the skin. Importantly, at a higher concentration (10 µg/10 µl), loperamide reduced A- and C-fiber CAP to a similar extent. In A- and C- fibers the conduction block produced by loperamide (5 ug/10 ul) was not prevented by naloxone pretreatment. This result is consistent with previous studies reporting a naloxone-resistant conduction block in peripheral nerve fibers by high concentrations of opioids. Morphine has previously been found to inhibit sodium and potassium currents when applied in high concentrations to the node of Ranvier of myelinated fibers in the frog sciatic nerve and these effects were naloxone insensitive [52]. Consistent with this finding, opioids (ethylmorphine, codeine, dihydrocodeine, morphine) at high concentrations inhibit myelinated fiber CAPs of the frog sciatic nerve in an opioid receptor independent mechanism [51]. Similarly, in single nerve fiber recordings, high concentrations of morphine (>2 mM) or naloxone (1 mM) blocked conduction in unmyelinated C-fibers [50]. The opioid meperidine (at 705 µM, i.e. 2 µg/10 µl) blocked conduction in myelinated and unmyelinated dorsal root fibers in a naloxone resistant manner [53], and it was found to blocked sodium channels similar to lidocaine [54]. Furthermore, high concentrations of fentanyl and sufentanil have previously been reported to decrease the CAPs in myelinated and unmyelinated fibers [55] (but also see [56]). Taken together, the results from previous and the current study suggest that loperamide may have local anesthetic activity at the doses/concentrations used in this study. Loperamide is known to block voltage-dependent calcium channels [57] and hyperpolarization-activated cyclic nucleotide-gated channels [58], [59], but it actions on sodium channels are currently unknown.

The naloxone *in vivo* experiments and the *in vitro* experiments were only performed in non lesioned animals or on nerves from unlesioned animals, respectively. Nerves from lesioned animals are in a pathological state, for example, due to the inflammatory response accompanying ongoing Wallerian degeneration. However, it is unlikely that naloxone or *in vitro* experiments on lesioned animals/nerves would have provided a different result as the inhibitory effect of loperamide on the mechanosensitivity of unmyelinated afferents was seen in both, lesioned and unlesioned animals. Importantly, the dose-dependency and the observed effect size were similar in both groups.

In our previous study [18], systemic and intraplantar administration of loperamide was anti-allodynic in the SNL model of neuropathic pain. The anti-allodynic effect of systemic loperamide was blocked by systemic pretreatment with methyl-naltrexone, a peripherally acting MOR-preferring antagonist, and by ipsilateral intraplantar pretreatment with the highly selective MOR antagonist CTAP. These findings are in conflict with the results reported here, as naloxone pretreatment did neither prevent the loperamide induced inhibition of mechanosensitivity nor the observed conduction block in primary afferents. However, following intraplantar injection, loperamide may exert two effects: a naloxone resistant local anesthetic effect and a naloxone sensitive, antihyperalgesic effect. In agreement with this hypothesis, we recently observed that naltrexone pretreatment did not affect the reversal of spinal nerve injury- induced heat hyperalgesia 10–15 min following loperamide injection. However, naltrexone (but not vehicle) pretreatment reversed the loperamide effects 45–60 min after loperamide injection (Chung et al., submitted). In our previous study [18], animals were tested 40–60 minutes following loperamide injection. A recent study on CFA injured animals, however, found that naloxone-methiodide (100 µg/100 µl) coinjected intraplantar with loperamide (100 ug/100 µl) prevented the increase in paw pressure threshold seen in CFA animals injected intraplantar with loperamide [60]. We do not know what explains this different finding.

### Comparison of Intraplantar and Systemic Administration of Loperamide

Before attributing the systemic effects of systemic loperamide to a peripheral blockade of nociceptor input similar to that observed in this study with intraplantar loperamide injections, we need to consider the doses used. The effective intraplantar dose in the current studies (−5 µg/10 µl) is several orders of magnitude higher that the systemic dose used in our previous studies (ED_50_ = 0.78 mg/kg . 7.8 ng/10 µl). In the present study intraplantar doses of 1.5 µg/10 µl were not effective. Unlike local loperamide, systemic loperamide did not inhibit the mechanical response of unmyelinated nociceptors. These observations suggest that the mechanisms underlying the pain relief of systemic loperamide may be different from those for intraplantar injection. One possibility is that low doses of systemic loperamide attenuate ectopic, spontaneous activity in nociceptors without affecting mechanical sensitivity under neuropathic conditions. Spontaneous activity in injured and in adjacent uninjured afferents following an SNL lesion has been proposed to lead to central sensitization and to the development of mechanical hypersensitivity [26], [61], [62]. In an animal neuroma model, systemic lidocaine reduced spontaneous activity originating from the neuroma and dorsal root ganglion at doses below those producing conduction block [63]. Similarly, spontaneous activity and behavioral signs of mechanical allodynia are attenuated by drugs that inhibit sodium channel activity, including tricyclic antidepressants, anticonvulsants [64] and fluphenazine, an antipsychotic drug [65]. In a human model of electrically evoked pain and hyperalgesia, alfentanil (and lidocaine) reduced not only pain, allodynia and hyperalgesia but also the accompanying skin flare [66] suggesting a peripheral effect on unmyelinated C fiber function. It is therefore likely, that an anesthetic, i.e. membrane-stabilizing effect of loperamide contributes to the antihyperalgesic effect that we have observed previously following intraplantar and systemic injection of loperamide in a behavioral model of neuropathic pain [18].
